# Volumetric intensity-modulated Arc (RapidArc) therapy for primary hepatocellular carcinoma: comparison with intensity-modulated radiotherapy and 3-D conformal radiotherapy

**DOI:** 10.1186/1748-717X-6-76

**Published:** 2011-06-21

**Authors:** Yu-Cheng Kuo, Ying-Ming Chiu, Wen-Pin Shih, Hsiao-Wei Yu, Chia-Wen Chen, Pei-Fong Wong, Wei-Chan Lin, Jeng-Jong Hwang

**Affiliations:** 1 Dept. of Biomedical Imaging & Radiological Sciences, National Yang-Ming University, No. 155, Sec. 2, Li-Nong St., Bei-tou, Taipei 11221, Taiwan; 2 Dept. of Radiation Oncology, China Medical University Hospital, No. 2, Yuh-Der Rd. Taichung, 404, Taiwan; 3 Dept. of Anesthesiology, China Medical University Hospital, No. 2, Yuh-Der Rd. Taichung, 404, Taiwan; 4 Dept. of Biomedical Imaging & Radiological Sciences, China Medical University, No. 2, Yuh-Der Rd. Taichung, 404, Taiwan; 5 Graduate Institute of Epidemiology, National Taiwan University, 5F, No.17, Hsu-Chow Rd. Taipei, 100, Taiwan; 6 Dept. of Radiation Oncology, Wan-Fang Hospital, No. 111, Section 3, Hsing-Long Rd. Taipei, 116, Taiwan; 7 Dept. of Radiation Physics, The University of Texas MD Anderson Cancer Center, 1515 Holcombe Bd. Unit No. 94, Houston, TX 77030, USA

## Abstract

**Background:**

To compare the RapidArc plan for primary hepatocellular carcinoma (HCC) with 3-D conformal radiotherapy (3DCRT) and intensity-modulated radiotherapy (IMRT) plans using dosimetric analysis.

**Methods:**

Nine patients with unresectable HCC were enrolled in this study. Dosimetric values for RapidArc, IMRT, and 3DCRT were calculated for total doses of 45~50.4 Gy using 1.8 Gy/day. The parameters included the conformal index (CI), homogeneity index (HI), and hot spot (V_107%_) for the planned target volume (PTV) as well as the monitor units (MUs) for plan efficiency, the mean dose (D_mean_) for the organs at risk (OAR) and the maximal dose at 1% volume (D_1%_) for the spinal cord. The percentage of the normal liver volume receiving ≥ 40, > 30, > 20, and > 10 Gy (V_40 Gy_, V_30 Gy_, V_20 Gy_, and V_10 Gy_) and the normal tissue complication probability (NTCP) were also evaluated to determine liver toxicity.

**Results:**

All three methods achieved comparable homogeneity for the PTV. RapidArc achieved significantly better CI and V_107% _values than IMRT or 3DCRT (*p *< 0.05). The MUs were significantly lower for RapidArc (323.8 ± 60.7) and 3DCRT (322.3 ± 28.6) than for IMRT (1165.4 ± 170.7) (*p *< 0.001). IMRT achieved a significantly lower D_mean _of the normal liver than did 3DCRT or RapidArc (*p *= 0.001). 3DCRT had higher V_40 Gy _and V_30 Gy _values for the normal liver than did RapidArc or IMRT. Although the V_10 Gy _to the normal liver was higher with RapidArc (75.8 ± 13.1%) than with 3DCRT or IMRT (60.5 ± 10.2% and 57.2 ± 10.0%, respectively; *p *< 0.01), the NTCP did not differ significantly between RapidArc (4.38 ± 2.69) and IMRT (3.98 ± 3.00) and both were better than 3DCRT (7.57 ± 4.36) (*p *= 0.02).

**Conclusions:**

RapidArc provided favorable tumor coverage compared with IMRT or 3DCRT, but RapidArc is not superior to IMRT in terms of liver protection. Further studies are needed to establish treatment outcome differences between the three approaches.

## Background

Hepatocellular carcinoma (HCC) is the fifth most common malignancy and the third most common cause of cancer-related death in the world [1]. Surgical resection has been proven as the major treatment modality for HCC. However, most patients with HCC have unresectable disease at diagnosis. These patients are treated with other treatment modalities, such as percutaneous ethanol injection (PEI), radiofrequency ablation (RFA) therapy, transcatheter arterial chemoradiotherapy (TACE), or sorafenib, but the response to treatment is limited [2-6].

The use of radiation therapy (RT) for the treatment of HCC was first investigated more than 40 years ago, but the early trials reported poor results due to the low tolerance of the whole liver to radiation and severe hepatic toxicity, or radiation-induced liver disease (RILD) caused by whole liver irradiation [7,8]. RILD, a clinical syndrome characterized by ascites, anicteric hepatomegaly, and impaired liver function, is developed in 5% of patients who received 30~33 Gy whole liver irradiation and usually occurs 2 weeks to 4 months after completion of RT. RILD usually resolves after supportive care. Unfortunately, severe RILD may develop into hepatic failure and even death [9,10]. The low hepatic tolerance to radiation also limits the application of higher radiation doses to the tumor. In 1991, Emami et al. reported that the TD_5/5 _(the tolerance dose leading to a 5% complication rate at 5 years) for 1/3, 2/3, and the whole liver at 1.8~2 Gy/day were 50 Gy, 35 Gy, and 30 Gy, respectively [11]. Dawson *et al *used the normal tissue complication probability (NTCP) of the Lyman model to describe the relationship between irradiated liver volume and radiation dose and they demonstrated that a higher radiation dose could be delivered safely to liver tumors, with better outcomes, if only part of the liver was irradiated [12]. As image-based treatment planning and engineering has advanced, three-dimensional conformal radiotherapy (3DCRT) was developed to irradiate the tumor accurately while minimizing the dose to the normal liver. A number of studies have demonstrated encouraging results showing that a radiation dose could be safely increased to part of the liver using 3DCRT [13]. For example, Park et al. reported a significant relationship between the total dose to the liver tumor and the tumor response (< 40 Gy, 40-50 Gy, and > 50 Gy giving responses of 29.2%, 68.6%, and 77.1%, respectively) without significant toxicity (rate of liver toxicity: 4.2%, 5.9%, and 8.4%, respectively).

Despite improvements to 3DCRT, dose distribution remains suboptimal in some cases. In the early 2000s, the development of inverse planning systems and multileaf collimators (MLCs) culminated in a more sophisticated technique, intensity-modulated radiotherapy (IMRT). Using an inverse planning algorithm to generate multiple nonuniform-intensity beams, IMRT can potentially deliver a higher dose to the tumor while delivering a relatively lower dose to the normal liver as compared with 3DCRT. Cheng *et al*. suggested that IMRT might be able to preserve acceptable target coverage and potentially reduce NTCP values (IMRT = 23.7% and 3DCRT = 36.6%, *p *= 0.009) compared with 3DCRT [14]. Fuss et al. reported that IMRT allowed a dose escalation to 60 Gy, in which range 3DCRT had to reduce the total dose to decrease the probability of RILD to acceptable levels [15].

The RapidArc technique, developed by Varian Medical Systems about 2 years ago, is a volumetric intensity-modulated arc therapy that accurately and efficiently delivers a radiation dose to the target using a one-or two-arc gantry rotation by simultaneously modulating the MLC motion and the dose rates. RapidArc has been shown to be equivalent or superior to IMRT for some malignancies, including head and neck cancer and prostate cancer [16-18], but there has been no study to determine the feasibility of using RapidArc for the treatment of primary HCC. The purpose of our study was to compare the RapidArc radiation treatment plans for patients with HCC with 3DCRT and IMRT plans using dosimetric analysis. The PTV coverage and critical organ sparing for each technique were determined using dose-volume histograms (DVH) and the NTCP model.

## Methods

### Patient Characteristics

From April 2008 to July 2010, we enrolled nine patients who had primary HCC diagnosed at China Medical University Hospital. All patients underwent alpha-fetoprotein (AFP) examination, contrast-enhanced computed tomography (CT), and ultrasonography to confirm the diagnosis. All patients were male and the median age was 57 years (range, 38-81 years). Five patients had Child-Pugh score A cirrhosis and 4 had Child-Pugh score B cirrhosis. Eight (88.9%) patients had American Joint Committee on Cancer (6^th ^edition) stage III disease, and 1 (11.1%) patient had stage IV disease.

### Immobilization, Simulation, and Target Delineation

The patients were immobilized using vacuum casts in a supine position with both arms raised above their heads. Non-contrast CT simulation was performed with a 5-mm slice thickness and included whole liver and bilateral kidney scans. Respiratory control and abdominal compression were not used. After simulation, the CT images were transferred into the Eclipse treatment planning system (Version 8.6.15, Varian Medical System, Inc., Palo Alto, CA, US), and target delineation was performed with the aid of the contrast-enhanced CT images.

We defined the gross tumor volume (GTV) as the volume of primary tumor evident on contrast-enhanced CT images. The clinical target volume (CTV) was delineated on the basis of the GTV expanded by 5 mm. The planning target volume (PTV) was defined as the CTV with a 5-mm radial expansion and a 10-mm craniocaudal expansion to account for errors caused by the daily setup process and internal organ motion. The normal liver volume was defined as the total liver volume minus the GTV. All of the contours were drawn by the same physician.

### Treatment Planning and Dose Delivery

In our study, we prescribed 95% of total dose to cover ≥ 95% of the PTV coverage in daily 1.8-Gy fractions while keeping the minimum dose ≥ 93% of total dose and maximum dose ≤ 107% of total dose and normalized all plans to the mean dose of PTV. The guidelines for dose prescription were as follows. When the normal liver volume irradiated with > 50% of the isocenter dose was < 25%, 25-50%, or 50-75%, the total dose prescribed was > 59.4 Gy, 45-54 Gy, and 41.4 Gy, respectively [19]. No patient received whole liver irradiation. The constraints for the organs at risk (OARs), can be seen in Table 1. These were imposed in terms of the TD_5/5 _to ensure that the maximal tolerated doses to the normal liver, stomach, kidneys, and spinal cord were not exceeded [11]. Six-or 10-MV photon beams were used, depending on the tumor location, and the same energy was used for each patient and for all three methods.

**Table 1:** The dose constraints of organ at risk

OAR	Dose constraints
Normal liver	Mean dose ≤ 26 Gy
Stomach	Maximum dose ≤ 54 Gy
Kidney	At least one side of kidney ≤ 23 Gy (mean dose)
Spinal cord	Maximum dose ≤ 47 Gy (Maximum dose of spinal cord plus 5-mm margin ≤ 45 Gy)

OAR: Organ at risk.

For each patient, three different plans (3DCRT, IMRT, and RapidArc) were calculated using the Eclipse planning system with the 120-leaf multi-leaf collimator (MLC) (Varian Medical Systems). For the 3DCRT and IMRT plans, all the gantry angles and numbers of radiation fields (range, 4-5) were manually selected on the basis of the morphological relationship between the PTV and OARs to cover at least 95% of the PTV and spare the OARs. A dose rate of 400 MU/min was used. For RapidArc, the plans were optimized using the two-arc technique with gantry angle running counterclockwise from 179° to 181° and clockwise from 181° to 179° and with the dose rate varied between 0 MU/min and 600 MU/min (upper limit). The optimization constraints for OARs using RapidArc were the same as the constraints in Table 1.

### Plan Evaluation

1. PTV coverage

The dose to the PTV was evaluated using DVHs with the following parameters:

a. V_x% _means the volume receiving ≥ x% of the prescribed dose. For example, the V_100% _of the PTV was used to prescribe the PTV coverage, and V_107% _was used to represent the hot spot in the PTV.

b. The conformity index (CI) = (V_PTV_/TV_PV_)/(TV_PV_/V_TV_) = V_PTV _× V_TV_/TV_PV_^2^, where V_PTV _is the volume of the PTV, TV_PV _is the portion of the V_PTV _within the prescribed isodose line, and V_TV _is the treated volume of the prescribed isodose line [17,20]. The CI represented the dose fit of the PTV relative to the volume covered by the prescribed isodose line. The smaller and closer the value of CI is to 1, the better the conformity of the PTV.

c. The homogeneity index (HI) = D_5%_/D_95%_, where D_5% _and D_95% _are the minimum doses delivered to 5% and 95% of the PTV [17,21]. HI is a ratio that is used to evaluate the homogeneity of the PTV. The smaller and closer the value of HI is to 1, the better the homogeneity of the PTV.

2. OARs sparing

a. V_nGy _is the percentage of organ volume receiving ≥ n Gy. In this study, V_40 Gy _was the percentage of the normal liver volume receiving ≥ 40 Gy, which represents high-dose exposure for the normal liver. In contrast, V_10 Gy _was the percentage of the normal liver volume receiving ≥ 10 Gy, which represented low-dose exposure for the normal liver.

b. We used the normal tissue complication probability (NTCP), from the Lyman model, to measure the probability of RT complications in the normal liver [22]. In the NTCP model,(1)(2)

where *EUD *is the equivalent uniform dose, converted from the dose-volume pairs [*D_i_, v_i_*], to describe the dose which, if delivered uniformly to the entire organ, would achieve the same effect as the given heterogeneous dose distribution demonstrated by the DVH. The TD_50_(1) is the dose to the whole liver that would result in a 50% probability of toxicity. The parameter "m" is the steepness of the dose-complication curve for a fixed partial volume. The parameter "n" is the slope of the complication probability, which determines the dose-volume relationship for the irradiated normal liver. In this study, the following values for the parameters were used: n = 0.32, m = 0.15, and TD_50_(1) = 40 Gy [23].

### Statistical Analyses

The dosimetric differences among the three treatments for the nine patients were analyzed using the Friedman test. When a significant difference (*p *< 0.05) was found, the difference between two treatments for each effect was further examined by Wilcoxon signed-rank test. All analyses were performed using SPSS software, version 15.0 (SPSS Inc., Chicago, IL).

## Results

### PTV Coverage, CI, and HI

**Figure 1:**
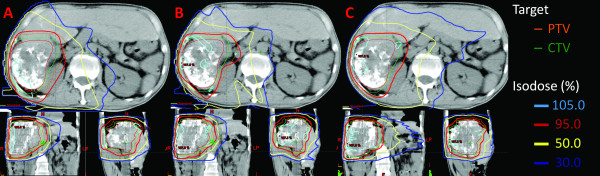
**The comparison of isodose distributions of PTV and OAR in 3DCRT, IMRT and RapidArc**. A: 3DCRT, B: IMRT and C: RapidArc. RapidArc achieved better conformality to the 95% isodose line (red line) of the PTV and better spinal cord sparing to the 50% isodose line (yellow line) as compared with 3DCRT and IMRT. However, RapidArc obtained higher 30%-isodose coverage (blue line) of volume of the normal liver than did 3DCRT and IMRT.

**Figure 2:**
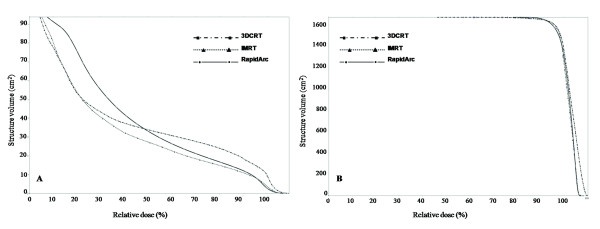
**The comparison of DVHs for PTV and normal liver in 3DCRT, IMRT and RapidArc**. Right figure = DVHs of PTV. These three techniques produced similar homogeneity of the PTV. Left figure = DVHs of normal liver. RapidArc obtained the higher low-dose distribution in the normal liver compared with 3DCRT and IMRT. On the other hand, 3DCRT obtained the high-dose distribution in the normal liver compared with IMRT and RapidArc.

The mean gross tumor volume (GTV) was 979.3 ± 497.2 cm^3 ^(range, 346.5-2019.3 cm^3^). The mean planned tumor volume (PTV) was 1734.2 ± 923.0 cm^3 ^(range, 859.6-3253.4 cm^3^). The mean normal liver volume was 1632.4 ± 539.0 cm^3 ^(range, 933.7-2270.6 cm^3^). None of the PTVs included the whole liver. The prescribed total dose was 49.4 ± 1.9 Gy (range, 45-50.4 Gy). The dose rate of RapidArc varied between 0 MU/min and 461 MU/min. The typical dose distributions and dose-volume histograms (DVH) for PTV and OARs are shown in Figure 1 and 2, respectively. In Figure 1C, RapidArc achieved better conformality to the 95% isodose line of the PTV than did 3DCRT and IMRT. In addition, RapidArc also achieved better spinal cord sparing to the 50% isodose line than did 3DCRT and IMRT. However, RapidArc resulted in higher coverage at the 30% isodose line in the normal liver as compared with 3DCRT (Figure 1A) or IMRT (Figure 1B), which means higher low-dose exposure occur for the normal liver with RapidArc. In Figure 2, the right DVH showed that all of the PTVs were fixed between V_95% _and V_107%_, without any significant differences. The left DVH showed that the low-dose distribution in the normal liver was greater for RapidArc than for 3DCRT or IMRT, and the high-dose distribution was greater for 3DCRT than for IMRT or RapidArc.

Table 2 summarizes the results for the investigated DVH-parameters, including CTV coverage, PTV coverage, monitor unit (MU) dose and OAR dose for the 9 patients. Table 3 shows the differences among the three methods with regard to the DVH parameters. For target coverage, all V_95% _of CTV for these three techniques gave at least 99% of the prescribed dose without any significant difference (*p *= 1.00). For the PTV coverage, the mean CI of RapidArc (1.12 ± 0.05) was significantly lower than that of IMRT (1.19 ± 0.06) and 3DCRT (1.286 ± 0.11) (*p *< 0.05). The V_95%_, and V_100% _valus for PTVs and HI were 95.50 ± 2.41, 76.81 ± 5.95 and 1.13 ± 0.05 (3DCRT), 95.27 ± 1.99, 77.88 ± 4.27 and 1.13 ± 0.04 (IMRT), and 95.31 ± 1.64, 77.47 ± 2.64 and 1.12 ± 0.03 (RapidArc), respectively, with no significant differences among methods (*p *= 1.00, 1.00 and 0.69, respectively). For the hot spot sparing, the mean V_107% _of the PTV was significantly highest for 3DCRT (7.49 ± 7.92) and the lowest was RapidArc (1.74 ± 2.82); this indicates that there was better hot-spot sparing of the PTV with RapidArc than with IMRT or 3DCRT (*p *< 0.05).

**Table 2:** The summary of all investigated DVH-parameters as mean values ± standard deviation (SD)

		3DCRT	IMRT	RA
CTV	V_95% _(%)	99.57 ± 0.39	99.65 ± 0.42	99.69 ± 0.42
PTV	V_95% _(%)	95.50 ± 2.41	95.27 ± 1.99	95.31 ± 1.64
	V_100% _(%)	76.81 ± 5.95	77.88 ± 4.27	77.47 ± 2.64
	V_107% _(%)	7.49 ± 7.92	3.71 ± 3.00	1.74 ± 2.82
	CI	1.286 ± 0.11	1.19 ± 0.06	1.12 ± 0.05
	HI	1.13 ± 0.05	1.13 ± 0.04	1.12 ± 0.03
Normal liver	D_mean _(Gy)	21.58 ± 3.01	19.31 ± 2.89	21.97 ± 2.61
	V_40 Gy _(%)	23.05 ± 4.06	18.61 ± 4.13	18.85 ± 3.97
	V_30 Gy _(%)	32.10 ± 6.80	26.23 ± 5.87	27.77 ± 5.34
	V_20 Gy _(%)	42.12 ± 7.56	37.16 ± 8.65	43.67 ± 8.18
	V_10 Gy _(%)	60.55 ± 10.24	57.24 ± 10.02	75.77 ± 13.13
	NTCP	7.57 ± 4.36	3.98 ± 3.00	4.38 ± 2.69
Stomach	D_mean _(Gy)	23.16 ± 16.50	20.63 ± 15.26	23.42 ± 13.70
Left Kidney	D_mean _(Gy)	11.37 ± 6.62	8.36 ± 4.60	7.69 ± 5.06
Right Kidney	D_mean _(Gy)	14.99 ± 13.11	13.11 ± 11.42	11.84 ± 10.41
Spinal Cord	D_1% _(Gy)	38.94 ± 7.62	43.89 ± 2.01	38.51 ± 8.90
MU		322.33 ± 28.62	1165.44 ± 170.68	323.78 ± 60.65

PTV: planned tumor volume; MU: monitor unit; 3DCRT: 3-D conformal radiation therapy; IMRT: intensity-modulated radiation therapy; RA: RapidArc.

**Table 3:** All differences among three methods with regard to the DVH-parameters

	P value			
	
	Overall	IMRT vs 3DCRT	IMRT vs RA	RA vs 3DCRT
CTV				
V_95% _(%)	1.00	--	--	--
PTV				
V_95% _(%)	1.00	--	--	--
V_100% _(%)	1.00	--	--	--
V_107% _(%)	0.016	--	RA < IMRT *	RA < 3DCRT *
CI	0.004	IMRT < 3DCRT *	RA < IMRT *	RA < 3DCRT *
HI	0.69	--	--	--
				
Normal liver				
D_mean _(Gy)	0.001	IMRT < 3DCRT *	IMRT < RA *	--
V_40 Gy _(%)	0.004	IMRT < 3DCRT **	--	RA < 3DCRT *
V_30 Gy _(%)	0.004	IMRT < 3DCRT **	--	RA < 3DCRT *
V_20 Gy _(%)	0.004	IMRT < 3DCRT **	IMRT < RA *	--
V_10 Gy _(%)	0.007	--	IMRT < RA **	3DCRT < RA *
NTCP	0.002	IMRT < 3DCRT **	--	RA < 3DCRT *
Stomach D_mean _(Gy)	0.121	IMRT < 3DCRT *	--	--
Left Kidney D_mean _(Gy)	0.085	IMRT < 3DCRT *	--	--
Right Kidney D_mean _(Gy)	0.217	--	--	--
Spinal Cord D_1% _(Gy)	0.236	--	--	--
MU	0.001	3DCRT < IMRT **	RA < IMRT **	--

p < 0.05; ** p < 0.01

PTV: planned tumor volume; V_x%_: the volume receiving ≥ x% of the prescribed dose; V_nGy_: the percentage of organ volume receiving ≥ n Gy; CI: conformity index; HI: homogeneity index; D_mean_: the mean dose for the organ; D_1%_: the maximal dose at 1% volume for the organ; MU: monitor unit; 3DCRT: 3-D conformal radiation therapy; IMRT: intensity-modulated radiation therapy; RA: RapidArc.

### OARs Sparing

The mean doses to the normal liver for each method were 21.58 ± 3.01 Gy (3DCRT), 19.31 ± 2.89 Gy (IMRT), and 21.97 ± 2.61 Gy (RapidArc), with a significantly lower mean dose to the normal liver with IMRT than with 3DCRT or RapidArc (*p *< 0.05). The high-dose regions of the normal liver were higher for V_40 Gy _and V_30 Gy _with 3DCRT (23.05 ± 4.06 and 32.10 ± 6.80) than with IMRT (18.61 ± 4.13 and 26.23 ± 5.87) (*p *< 0.01) or RapidArc (18.85 ± 3.97 and 27.77 ± 5.34) (*p *< 0.05). The low-dose region of the normal liver was higher for V_10 Gy _with RapidArc (75.77 ± 13.13) than with IMRT (57.24 ± 10.02) (*p *< 0.01) or 3DCRT (60.55 ± 10.24) (*p *< 0.05). In Table 3, the NTCP value for 3DCRT (7.57 ± 4.36) was significantly higher than that for IMRT (3.98 ± 3.00) (*p *< 0.01) or RapidArc (4.38 ± 2.69) (*p *< 0.05), but there was no significant difference in the NTCP between IMRT and RapidArc (*p *= 0.26). For the other OARs, there were no significant differences in dose among the three methods, except for a lower mean dose to the stomach and left kidney, respectively, with IMRT (20.63 ± 15.26 Gy and 8.36 ± 4.60 Gy) than with 3DCRT (23.16 ± 16.50 Gy and 11.37 ± 6.62 Gy) (*p *< 0.05). The maximum dose to the spinal cord (D_1%_) was equal for all three methods.

### Efficiency Analysis

IMRT had three times the MUs (1165.44 ± 170.68) of RapidArc (323.78 ± 60.65) and 3DCRT (322.33 ± 28.62) (*p *< 0.01). There was no significant difference in the numbers of MUs between 3DCRT and RapidArc (*p *= 0.859).

## Discussion

Historically, the role of RT in HCC has been limited because of the risk of RILD caused by whole liver irradiation. Improved knowledge of partial liver RT has created renewed in using RT with HCC and, furthermore, technical advancements in 3DCRT have allowed higher doses to targeted to the tumors while minimizing exposure of surrounding liver tissue. Recently, more and more types of conformal RT have been developed to deliver highly conformal treatment with minimal damage to surrounding normal liver parenchyma, including IMRT, image-guided radiotherapy (IGRT) and stereotactic body radiotherapy (SBRT) [24]. RapidArc is a novel form of volumetric intensity-modulated RT that has the advantages of a short treatment time, fewer MUs and the availability of highly conformal treatment plans. Several investigations have demonstrated the advantages of RapidArc. Verbakel *et al*. demonstrated that RapidArc achieved similar PTV coverage and OAR sparing but lower MUs than IMRT in patients with head and neck cancers. Besides, double arc plans yielded better PTV coverage than single arc or IMRT [16]. Palma *et al*. reported that variable dose rate volumetric modulated arc therapy achieved better dose distribution and lower MUs than IMRT in patients with prostate cancers. This work was a pilot study to investigate the dosimetric difference of a RapidArc plan for HCC compared to 3DCRT and IMRT plans.

In our study, the homogeneity of the PTV provided by all three techniques was similar, but the RapidArc was able to achieve better conformity and hot-spot sparing of the PTV compared to IMRT or 3DCRT (*p *< 0.05). For OARs sparing, the three methods showed comparable results in terms of the mean dose to the stomach and kidneys and maximum dose to the spinal cord. For the normal liver, 3DCRT provided the worst dose distribution, with significantly worse D_mean_, V_40 Gy_, V_30 Gy_, and NTCP values than RapidArc or IMRT. Compared with IMRT, RapidArc provided comparable V_40 Gy_, V_30 Gy_, and NTCP values for the normal liver, but RapidArc achieved significantly higher D_mean_, V_20 Gy _and V_10 Gy _values for the normal liver.

The Lyman NTCP model has been widely used to predict or estimate the probability of normal tissue complication. This model supposed there is a sigmoid relationship between a uniform radiation dose given to a part of the volume in an organ and the probability of complication. More and more authors have used this model to predict RILD. Burman *et al*. assigned the parameters to be as follows, n as 0.32, m as 0.15, and TD_50_(1) as 40 Gy, in a model that predict the risked of RILD [23]. Cheng *et al*. applied the values of n = 0.35, m = 0.35 and TD_50_(1) = 49.4 Gy in another model [25]. Dawson *et al*. further modified the parameter TD_50_(1) to 39.8 Gy for hepatobiliary cancer and to 45.8 Gy for liver metastasis (n = 0.97 and m = 0.12) [26]. Although different values for the parameters have been applied to the Lyman NTCP model by different authors, they demonstrated the feasibility of partial liver irradiation. If the TD_50 _is kept constant, the NTCP value is represented as a function of partial volume. This organ demonstrates a "threshold type behavior" and the NTCP value will rise only if a certain volume is irradiated. Furthermore, the NTCP value of the partial volume depends on the dose. Therefore, we believe that the V_40 Gy _and V_30 Gy _influence the NTCP values of the normal liver more than V_20 Gy _and V_10 Gy _do. In addition, Dawson *et al*. also addressed whether those who had impaired liver function might not be suitable for the Lyman NTCP model and whether a better understanding of the mechanism of RILD may improve the accuracy of Lyman model in the future.

In addition to value used for NTCP, the V_30 Gy _and D_mean _of the normal liver play important roles in predicting the risk of RILD. Dawson *et al*. demonstrated that the D_mean _of normal liver was associated with the risk of RILD [26]. Yamada *et al*. reported a deterioration in the Child-Pugh Score in 5 out of 6 patients with a V_30 Gy _> 40%, *vs*. 2 of 13 patients with a V_30 Gy _< 40% (*p *< 0.01) [27].

Another issue that should be kept in mind is the higher low-dose irradiation to normal liver compared with 3DCRT or IMRT when RapidArc is used. Shueng *et al*. published a case of cholangiocarcinoma with bone metastasis who had received palliative RT for bone pain who developed radiation pneumonitis [28]. They demonstrated that, in this case, although the V_5 Gy _of the normal lung was only 20%, this still potentially induced radiation pneumonitis. One of the possible causes is an interaction between radiation-induced inflammation within the previously irradiated field and chemotherapy. Yamashita *et al*. has reported that the incidence of lung toxicity will become higher if large amount of low dose radiation is delivered [29]. In addition to the dosimetric impact, several investigators reported that some biological factors are associated with RILD. For example, Cheng *et al*. reported that the HBV carriers or cases with Child-Pugh B cirrhosis were correlated with the risk of RILD after 3D-CRT [25]. Xu *et al*. also reported that the Child-Pugh classification was associated with RILD [30]. In the current study, the potential risk of RILD caused by low-dose irradiation is unclear, but HCC patients in Taiwan usually have hepatitis B or C infection and liver cirrhosis and they usually received TACE, PEI or targeted therapy before RT. Radiation oncologists should be aware of the potential risk of higher low-dose exposure to the normal liver when RapidArc is used.

From the view of dosimetric comparison, one of the reasons that RapidArc is not better than IMRT for liver protection may be that HCC is always surrounded by normal liver parenchyma, which is the major concern when using the volumetric RapidArc technique. In our study, we found that RapidArc increased the V_10 Gy_, V_20 Gy _and D_mean _of the normal liver compared to IMRT and, therefore, we suggest that the RapidArc should be used more carefully when treating HCC cases even if both RapidArc and IMRT achieve equivalent V_30 Gy _for the normal liver and have similar NTCP values.

Another advantage of RapidArc over IMRT were the reduction in the number of MUs. Several studies have reported that the disadvantages of IMRT include higher MUs, longer delivery times, and more low-dose exposure of organs at risk (OARs), all of which increase the risk of a radiation-induced second malignancy. Hall reported that IMRT, as compared with 3DCRT, might double the incidence of solid cancers in long-term survivors, especially children [31]. Zwahlen studied the cancer risk after IMRT for cervical and endometrial cancer and reported that cumulative second cancer risks (SCR) relative to 3DCRT for 6-MV and 18-MV IMRT plans were +6% and +26%, respectively [32]. There is no sufficient data to demonstrate that the lower MUs associated with RapidArc might reduce the risk of radiation-induced second malignancy. Furthermore, radiation-induced second malignancy occurs only in those who have long-term survival after treatment. Xu *et al*. reported that the 5-year survival rate for HCC patients receiving TACE plus RT was only 13% with a median survival time of 18 months [33]. Thus this advantage of RapidArc may have little influence on the prevention of radiation-induced second malignancy for HCC patients. Verbakel WF *et al*. [16] and Wagner *et al*. [34] compared RapidArc with IMRT for different malignancies and concluded that the major advantages of RapidArc over IMRT were the lower MUs and the shorter treatment time, which can be beneficial to the reduction of intra-fractional movement, improving patient comfort, and higher patient throughput.

Although RapidArc has been demonstrated the advantages on the treatment of other kinds of malignancies, the dosimetric advantage of RapidArc in our study is not always better than IMRT. Therefore it is not convincing that IMRT should be replaced by RapidArc when treating HCC. The limitations of our study include small patient numbers, relatively coarse 5 mm-slice thickness and a lack of respiratory control or abdominal compression. These limitations would possibly cause some errors in the dose calculation and analysis. Clinical trials and long-term follow-up are required to draw more definite conclusions. Therefore, we suggest that if PTV conformity and percentages of NTCP, D_mean_, V_30 Gy _and V_10 Gy _of the normal liver are acceptable, RapidArc may be selected on the basis of fewer MUs. If PTV coverage is not adequate or each of the above parameters related to liver toxicity is too high with RapidArc, then IMRT should be used.

In conclusion, RapidArc obtained favorable tumor coverage compared with IMRT and both RapidArc and IMRT achieved significantly better quality in terms of treatment plan when compared with 3DCRT. However, RapidArc is not superior to IMRT for liver protection. Nonetheless, RapidArc is a new technique, and optimization of its algorithm is still in its early stages (about 2 years of clinical experience), whereas 3DCRT and IMRT have been well-investigated and routinely used for more than 10 years. It is expected that more comprehensive planning systems for RapidArc are being developed and these might advance the optimization process in the future.

## Competing interests

The authors declare that they have no competing interests.

## Authors' contributions

YCK and HWY contributed significantly to study design and concept. YCK also contributed to manuscript writing and study coordinator. YMC and CWC contributed to statistical analysis. WPS and WCL contributed significantly to the acquisition of data and optimization of treatment plans. PFW and JJH contributed to final revision of manuscript. All authors read and approved the final manuscript.
